# Physical activity is associated with a low prevalence of musculoskeletal disorders in the Royal Norwegian Navy: a cross sectional study

**DOI:** 10.1186/1471-2474-8-56

**Published:** 2007-07-02

**Authors:** Tone Morken, Nils Magerøy, Bente E Moen

**Affiliations:** 1 University of Bergen and UNIFOB Helse, Section for Occupational Medicine, Kalfarveien 31, N-5018 Bergen, Norway; 2 University of Bergen, Department of Public Health and Primary Health Care, Section for Occupational Medicine, Bergen, Norway

## Abstract

**Background:**

Despite considerable knowledge about musculoskeletal disorders (MSD) and physical, psychosocial and individual risk factors there is limited knowledge about physical activity as a factor in preventing MSD. In addition, studies of physical activity are often limited to either leisure activity or physical activity at work. Studies among military personnel on the association between physical activity at work and at leisure and MSD are lacking. This study was conducted to find the prevalence of MSD among personnel in the Royal Norwegian Navy and to assess the association between physical activity at work and at leisure and MSD.

**Methods:**

A questionnaire about musculoskeletal disorders, physical activity and background data (employment status, age, gender, body mass index, smoking, education and physical stressors) was completed by 2265 workers (58%) 18 to 70 years old in the Royal Norwegian Navy. Multiple logistic regression with 95% confidence intervals was used to assess the relationship between physical activity and musculoskeletal disorders.

**Results:**

A total of 32% of the workers reported musculoskeletal disorders often or very often in one or more parts of the body in the past year. The most common musculoskeletal disorders were in the lower back (15% often or very often), shoulders (12% often or very often) and neck (11% often or very often). After adjustment for confounders, physical activity was inversely associated with musculoskeletal disorders for all body sites except elbows, knees and feet.

**Conclusion:**

The one-year prevalence of musculoskeletal disorders among workers in the Royal Norwegian Navy was rather low. A physically active lifestyle both at work and at leisure was associated with fewer musculoskeletal disorders among personnel in the Royal Norwegian Navy. Prospective studies are necessary to confirm the cause and effect in this association.

## Background

Musculoskeletal disorders (MSD), defined as self-reported musculoskeletal symptoms [1], are common and may result in suffering among individuals and have economic effects on society [2-4]. Despite considerable knowledge about MSD and physical, psychosocial and individual risk factors [5-7] little is known about physical activity as a factor in preventing MSD. The definition of physical activity has varied between numerous investigators and has also resulted in a high degree of variability of measurable units [8]. Lifestyle physical activity is one expression used that includes all leisure, occupational or household activities that are at least moderate to vigorous in intensity and can be planned or unplanned activities that are part of everyday life [9].

Physical activity is often recommended for preventing several diseases, including MSD [10], and incontrovertible evidence indicates that regular physical activity contributes to preventing cardiovascular disease and other chronic conditions [11]. Review studies on dose-response relationship between physical activity and health conclude that several health parameters are related to the amount of physical activity in a graded fashion [11,12]. Guidelines on physical activity recommend longer duration of moderate-intensity physical activity to achieve the same health benefits as for vigorous intensive activities [13]. However, whether physical activity prevents MSD is still not clear, and studies of physical activity are often limited to either leisure activity or physical activity at work. A review study on worksite physical activity programmes concluded with positive effect on MSD [14]. On the other hand, studies on the association between leisure physical activity and MSD show inconsistent results [15]. Hoogendoorn et al [16] claimed no evidence for the effect of physical activity during leisure time on low back pain, whereas Vuori [17] concluded that leisure physical activity is effective in preventing low back pain. Sustained sporting activities seem to favourably affect neck and shoulder symptoms [18]. However, sporting activities and physical training are also sources of musculoskeletal injuries [19]. Musculoskeletal injuries are defined as any physical complaint sustained by a person as a result of sport training [20], and many of these might be included among self-reported MSD.

Physical fitness for duty is an important military medical component for readiness and an integral part of the fit and healthy force pillar of health protection [21]. Improved physical fitness might help prevent MSD. However, some studies conclude with a high prevalence of MSD among military personnel [22-24], and MSD are the most prevalent source of disability among United States Army soldiers and Navy personnel [21,25]. Several studies describe frequent musculoskeletal injuries caused by physical training among military personnel [26-29]. We could not find studies on the association between physical activity at work and at leisure and MSD among military personnel.

In the Royal Norwegian Navy the employees are allowed to perform physical exercise 2 hours per week in their workday hours. The employees are both military and civilians. The military personnel are required to pass an annual fitness test, whereas the other group is not [30]. The possibility for physical activity among the employees might have an impact on MSD. We hypothesized more physical activity to be associated with less MSD among the Navy personnel.

This study aimed:

• to determine the prevalence of self-reported MSD among military personnel and civilians in the Royal Norwegian Navy; and

• to assess the association between physical activity at work and at leisure and MSD.

## Methods

### Subjects

In 2002, all employees working in the Royal Norwegian Navy (3878 persons) were invited to answer a self-administered questionnaire about MSD as part of a study on work and health in the Navy. The name, address and National Insurance number were preprinted on the form. The questionnaire was returned directly to the research group at the University of Bergen. The questionnaire was completed by 2265 workers (58%) 18 to 70 years old. There were 1657 (74%) military personnel and 593 (26%) civilians.

### The questionnaire and variables

#### Musculoskeletal disorders

MSD were recorded according to a modification of the Standardized Nordic Questionnaire [1]. The questions about MSD were phrased as follows: "Have you had complaints (pain or discomfort) the past 12 months in your __?" The list included the neck, shoulders, elbows, hands, upper back, lower back, hips, knees and feet. A five-point response scale "never, seldom, sometimes, often, very often" was used. The frequency of symptoms as response options for low back pain has been tested to correspond well with the number of days with symptoms [31].

#### Physical activity

Information on physical activity was obtained for both work time and leisure time, based on a questionnaire used in several Norwegian surveys [32]. The amount of physical activity at work was measured with the following question: "How much physical activity have you had during work in the past year (average per week)?" Two scales were provided, (1) heavy activity with sweating and heavy breathing and (2) light activity without sweating and heavy breathing. The respondent was asked to tick off one of the following options for each scale: "none", "less than 1 hour", "1–2 hours" and "3 hours and more". The same question was repeated for leisure time. For physical activity, a scale ranging from 0 to 18 was created. Heavy activity with sweating and heavy breathing counted twice as much as light activity without sweating and heavy breathing. This scale was categorised into 0–4 as low activity, 5–12 as moderate activity and 13–18 as high activity.

#### Other variables

Background data included age, gender, height, weight, smoking status and education as well as physical stressors at work and at leisure. Education was categorised into: 9–12 years of education, 12–14, 14–16 and ≥16 years. Naval college years were included in the years of education, also when taken as supplementary education. Smoking was categorised into "smoker" and "non smoker". Body mass index (BMI) was calculated from height and weight (body weight in kg/(height in m)^2^) and categorised as defined by WHO: < 25 (only six had < 20), 25 to < 30 and ≥30 [33].

Physical stressors were determined by asking: "Have you in your work in the Navy now or previously been exposed to: a) heavy lifting; b) twisted positions; or c) working with arms above shoulder height?". The same questions were repeated for work and leisure outside the Navy. A five-point scale ranging from "never" to "very much" (0–4) was used for each question. The 6 physical stressors questions were transformed into a physical stressors index ranging from 0 to 24. The physical stressors index was characterised as very low for scores 0–4, low for 5–8, high for 9–12 and as very high for scores 13–24.

### Statistical methods

The MSD variables were dichotomized into often or very often (scores 4–5) and never, seldom or sometimes (scores 1–3). On the five-point scale from "never" to "very often", it has been suggested that workers who report MSD "often" or "very often" from one or more parts of the body should be classified as having significantly impaired health [34]. Descriptive statistics were performed to assess the prevalence of MSD.

To compare number of workers within each category of physical activity and number of workers with MSD, we used χ^2 ^tests and tested the linear by linear association between the two variables. Differences in MSD between military and civilian, age, BMI, smoking, education, physical stressors index and physical activity were compared using *t*-tests and Pearson χ^2 ^test. The results are reported as means or proportions.

To study the relationship between physical activity and MSD, multiple logistic regression analysis was carried out for each body region separately, adjusted for known confounders. The list of confounders included age [35], gender [36], BMI [35-37], smoking status [38] education [39] and physical stressors [5] in addition to employment status (military/civilian). The logistic regression analysis was repeated excluding the answers to the questions about light physical activity to see whether the estimates of the association between physical activity and MSD changed. This means that only the answers to heavy physical activity were included to calculate the physical scale. To be able to compare the estimates when and when not including light physical activity we kept the scale range from 0 to 18.

The analyses were performed using the SPSS 13.0 computer package. The significance limit was set at *P *= 0.05. The Regional Committee for Medical Research Ethics, Western Norway, cleared the study protocol, and the Norwegian Data Inspectorate approved the study.

## Results

### Characteristics of the study population

Similar to the mean age in the total Navy population, the mean age among the respondents was 38 years (Table 1). Among military men the mean age was 36 years (range 19–62), among military women 29 years (range 19–53) and among civilians 47 years for both men and women (range 18–70). Eleven per cent of the respondents were women, and the according number in the total Navy population was 12%. Twenty-seven per cent of the workers were smokers, and 57% of the workers had a BMI exceeding 25. On the 0 to 18 point scale, the military personnel reported more physical activity (mean = 9.6, median = 9.0) than the civilians (mean = 8.4, median = 8.0). Military personnel and civilians differed in physical activity at the same relative level both at work and at leisure (*t*-test, *P *< 0.001). Among all workers 92% had performed some heavy physical activity each week the past year. We analysed each question in the physical stressors index and found that civilians reported more twisted positions (Pearson χ^2^, *P *= 0.001) and work above shoulder height (Pearson χ^2^, *P *< 0.001) both in the Navy and outside the Navy, but not more heavy lifting.

**Table 1:** Background data and differences between military and civilians (missing not shown) (*n *= 2265).

	Total	Military personnel	Civilians	*P*
Gender				
Male (%)	2001 (88.9)	1550 (94.1)	438 (74.2)	
Female (%)	250 (11.1)	97 (5.9)	152 (25.8)	
Age, mean (years)	38.3	35.2	46.9	*P *< 0.001*
Physical activity, mean (0–18)	9.3	9.6	8.4	*P *< 0.001*
Physical stressors index (0–24)				*P *< 0.001**
Very low (0–4)	377 (17.0)	247 (15.1)	129 (22.8)	
Low (5–8)	815 (36.7)	655 (39.9)	156 (27.5)	
High (9–12)	693 (31.2)	519 (31.6)	169 (29.8)	
Very high (13–24)	338 (15.2)	220 (13.4)	113 (19.9)	
Body mass index, mean	25.9	25.8	26.1	*P *= 0.11*
Smoking				*P *= 0.002**
Non-smoker (%)	1638 (73.2)	1231 (74.9)	396 (68.2)	
Smoker (%)	601 (26.8)	412 (25.1)	185 (31.8)	
Education				*P *< 0.001*
9–12 years (%)	37 (1.8)	8 (0.5)	28 (5.1)	
12–14 years (%)	577(27.4)	194 (12.6)	376 (67.9)	
14–16 years (%)	1002 (47.5)	887 (57.6)	111 (20.0)	
> 14 years (%)	492 (23.3)	452 (29.3)	39 (7.0)	

*T-test ** Pearson χ^2 ^test

### Prevalence of musculoskeletal disorders in the Navy

**Figure 1:**
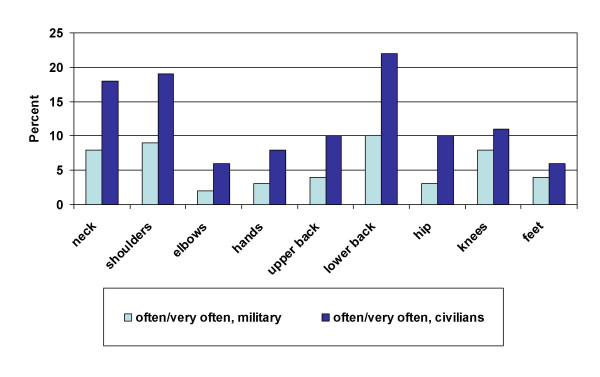
Musculoskeletal disorders experienced often or very often the past 12 months stratified by occupational status in the Royal Norwegian Navy (*n *= 2250).

A total of 85% of the employees in the Royal Norwegian Navy had experienced pain or other discomfort in one or more of the nine defined parts of the body during the past 12 months; 32% reported MSD often or very often in one or more parts of the body. The most common MSD were in the lower back (15% often or very often), shoulders (12% often or very often) and neck (11% often or very often). Civilians reported more MSD (often or very often) than military personnel in all parts of the body (Pearson χ^2^, *P *< 0.05) (Fig. 1). Women had more MSD (often or very often) than men in all parts of the body (Pearson χ^2^, *P *< 0.05) except for knees and feet (data not shown).

### Physical activity and musculoskeletal disorders

Table 2 shows the number of workers within each category of physical activity and the corresponding number with MSD often or very often from nine parts of the body. More physical activity was associated with a lower frequency of MSD. Test of the linear trend showed a significant association between physical activity and MSD in all parts of the body (P < 0.05), except for knees and feet.

**Table 2:** Workers within each category of physical activity and number (%) of workers with musculoskeletal disorders^† ^(*n *= 2258).

	Neck	Shoulders	Elbows	Hands	Upper back	Lower back	Hips	Knees	Feet
Physical activity *n *(%)	*n *(%)	*n *(%)	*n *(%)	*n *(%)	*n *(%)	*n *(%)	*n *(%)	*n *(%)	*n *(%)

Low 308 (100)	48 (16)	54 (18)	14 (5)	22 (8)	28 (10)	57 (19)	32 (11)	39 (13)	20 (7)
Moderate 1469 (100)	147 (10)	172 (12)	39 (3)	52 (4)	83 (6)	193 (13)	65 (5)	109 (8)	56 (4)
High 481 (100)	32 (7)	34 (7)	10 (2)	11 (2)	13 (3)	52 (11)	9 (2)	41 (9)	20 (4)

^† ^often or very often

In the logistic regression analysis (Table 3), physical activity was inversely associated with MSD often or very often in the neck, shoulders, hands, upper back, lower back and hips when adjusted for age, gender, BMI, smoking, education, employment status (military/civilian) and physical stressors. When the data on light physical activity were excluded from the analysis the odds ratios for the inversed associations between physical activity and MSD were slightly reduced: neck 0.94 (95% CI 0.91, 0.98), shoulders 0.96 (95% CI 0.93, 0.99), hands 0.96 (95% CI 0.91, 1.01), upper back 0.96 (95% CI 0.92, 1.00), lower back 0.97 (95% CI 0.94, 1.00) and hips 0.93 (95% CI 0.89, 0.98).

**Table 3:** Logistic regression analysis of the association between physical activity* and musculoskeletal disorders** by body parts^† ^(*n *= 2095)^‡^.

	Physical activity	Military/civilian^a^	Age	Gender^b^	BMI	Education	Physical stressors index	
	OR (95% CI)	OR (95% CI)	OR (95% CI)	OR (95% CI)	OR (95% CI)	OR (95% CI)	OR (95% CI)	
Neck	0.93 (0.89, 0.97)	1.58 (1.03, 2.42)	1.02 (1.00, 1.04)	3.26 (2.08, 5.11)	1.05 (1.01, 1.10)		High vs very low	2.33 (1.40, 3.88)
							Very high vs very low	4.27 (2.42, 7.53)
Shoulders	0.94 (0.91, 0.98)		1.03 (1.01, 1.04)	2.41 (1.57, 3.70)			High vs very low	
							Very high vs very low	2.42 (1.45, 4.02)
Elbows			1.03 (1.00,1.06)			0.56 (0.36, 0.86)	High vs very low	
							Very high vs very low	
Hands	0.93 (0.88, 0.99)		1.03 (1.01, 1.05)	2.99 (1.58, 5.65)			High vs very low	
							Very high vs very low	2.31 (1.01, 5.23)
Upper back	0.93 (0.88, 0.98)			3.07 (1.75, 5.40)			High vs very low	2.14 (1.11, 4.11)
							Very high vs very low	2.90 (1.37, 6.13)
Lower back	0.95 (0.92, 0.98)	1.51 (1.04, 2.19)	1.02 (1.01, 1.04)	1.56 (1.01, 2.43)			High vs very low	2.36 (1.48, 3.75)
							Very high vs very low	4.28 (2.56, 7.14)
Hips	0.90 (0.85, 0.95)		1.06 (1.03, 1.08)	2.57 (1.34, 4.93)			High vs very low	
							Very high vs very low	2.81 (1.28, 6.14)
Knees				1.80 (1.06, 3.05)	1.05 (1.00, 1.10)		High vs very low	
							Very high vs very low	2.46 (1.37, 4.42)

*Adjusted for employment category (military, civilian), age, gender, BMI (body mass index), smoking, education and physical stressors index. Smoking is not shown in the table as no significant associations were found.

** often or very often

^†^Feet are excluded from the table as no significant associations were found.

^‡^Non significant results are not shown.

^a^Military = 1, civilian = 2. ^b^Male = 1, female = 2

To illustrate how a change in the physical activity scale (including both light and heavy physical activity) would affect the odds ratio of MSD, we have calculated the following example: A difference in the physical activity scale of six points, such as 4 points versus 10 points, would mean the difference between a person with 1–2 hours of light physical activity both at work and at leisure compared with a person with an additional 3 hours of strenuous physical activity. In this case a higher physical activity would reduce the odds ratio of having MSD often or very often as follows: for the neck 36%, shoulders 30%, hands 34%, upper back 36%, lower back 28% and hips 48%. Physical activity was not associated with MSD (often or very often) in the elbows, knees and feet.

Being civilian versus military personnel was associated with more MSD (often or very often) in the neck and lower back (Table 3). Increasing age was associated with more MSD in the neck, shoulders, elbows, hands, lower back and hips. Women had more MSD in all parts of the body except the elbows and feet. Physical stressors were significantly and positively associated with MSD in all parts of the body except elbows and feet.

## Discussion

The study demonstrated a relationship between higher physical activity and less reported MSD. The significant associations were found between physical activity and reported MSD in all parts of the body, except elbows, knees and feet when adjusted for employment status, age, gender, BMI, smoking, education and physical stressors. Light physical activity strengthened the inverse association between physical activity and MSD.

A comparable study among aluminium workers in Norway also found a relationship between higher physical activity and less reported MSD without differentiating between light and heavy activity [40]. However, this study was limited to physical activity during leisure. The finding that light physical activity was associated with less MSD, is supported by studies on other health benefits [11]. A volume of exercise half the amount of physical activity that is currently recommended [13] may be of importance for several health outcomes. Anyway, since the majority of our study population performed heavy physical activity, the impact of light physical activity should be studied in a population performing predominantly light activity.

The fact that the significant association between higher physical activity and less MSD was present when adjusted for the physical stressors index supports the independent importance of physical activity versus physical stressors. A follow-up study by Leino-Arjas [41] found that high physical strenuousness at work increased the risk of later poor functioning, whereas physical activity at leisure was protective. Physical strenuousness could be compared with heavy lifting and working with the hands above the shoulders. The opposite effects of physical activity and physical strenuousness on functioning might be similar for the development of MSD, as our study indicates.

The lack of positive effect of physical activity on MSD in the knees and feet is supported by studies that demonstrate physical training as the cause of injuries in the lower limbs [27,28]. Athletic sports may increase the possibility of some lower-extremity disorders, and this might counteract the expected effect of physical activity on musculoskeletal health. A study on Australian Defence Force members found a high frequency of musculoskeletal problems, especially ankle, knee and spinal strains, among military personnel compared with a population in general practice [26]. Other studies have found high rates of military training-related injuries at or below the knee [28,29]. A literature study found some evidence that at least 4 hours per day of heavy physical activity increased the risk of osteoarthritis of the knees and running at least 20 miles (32 km) per week increased the risk of hip or knee osteoarthritis [17].

In the Navy, civilians had a higher prevalence of MSD than military personnel. An association between being a civilian and having MSD persisted for the neck and lower back when adjusted for age, gender, physical activity, BMI, smoking, education and physical stressors. The difference between civilians and military personnel might be explained by the selection of military personnel due to requirements for fitness for duty. The requirement of fitness among military personnel probably leads to a selection of healthier persons to the military group. Work factors not included in the analysis, such as psychosocial factors [5], might also differ among the two groups. This relationship should be studied further.

The one-year prevalence of 32% of frequent MSD in one or more parts of the body among workers in the Navy is rather low compared to other working populations [40,42,43]. In a study among aluminium workers in Norway using the same questionnaire on MSD, the comparable number was 49% [40]. The working populations both in the Navy and in the aluminium industry were dominated by men and had the same age. One explanation for the low prevalence of MSD in the Navy might be less physical risk factors in the work environment and more physical activity or exercise at work. Due to the demand of fitness test for military workers, this occupational group might differ from other working groups and these populations cannot be compared directly.

### Methodological considerations

The cross-sectional study design does not allow us to draw conclusions on a causal relationship between physical activity and MSD. It is not clear whether physical activity influences the MSD or, conversely, the MSD influence the amount of physical activity. On the one hand physical activity might result in less MSD, and on the other hand MSD might result in less physical activity. However, the study population consists of people at work, and people with severe and chronic pain unable to perform physical activity are probably very few. In any case, a hypothesis that physical activity prevents MSD should be followed up by prospective studies.

We are faced with selection bias at two levels. Firstly, the employees in the army might differ from other workers due to health certificate requirements (the healthy worker effect). The healthy worker effect has been shown to significantly influence results of cross-sectional studies dealing with low back pain [44]. Secondly, a selection bias due to the low response of 58% may be present if the non-responders had a different prevalence of MSD and a different degree of physical activity compared with those who responded. Similarly a different association between physical activity and MSD for the responders and non-responders could create such bias. However, the similar mean age and percentage of women and men among the respondents compared to the total Navy population may indicate that the respondents were representative for the Navy population. In addition, since the survey was part of a general health survey among employees and not specifically about MSD and physical activity we do not believe that the selection bias was a major issue.

Physical activity was measured by a questionnaire, and the validity and reliability of this method may be questionable [45]. However, a questionnaire may be the only feasible method of assessing physical activity in large populations. We asked about physical activity during work, and this might include physical activities that both benefit and harm musculoskeletal health. Anyway, physical stressors, like twisted positions and heavy lifting, was associated with more MSD, but higher physical activity was associated with less MSD, also when adjusted for the physical stressors index. More precise information on the type of activity could improve the validity for both the physical activity questions and the questions about physical stressors. Recall bias and social desirability bias can lead to misclassification of the amount of activity.

## Conclusion

The prevalence of MSD among workers in the Royal Norwegian Navy is relatively low compared with other working populations. A physically active lifestyle both at work and at leisure was associated with less MSD in most parts of the body among both military personnel and civilians. Prospective studies are necessary to confirm cause and effect in this association.

## Competing interests

The research programme Health, Safety and Work Environment in the Royal Norwegian Navy at the University of Bergen, Department of Public Health and Primary Health Care, Section for Occupational Medicine, Norway has been funded by the Royal Norwegian Navy, including the funding for some of the staff. The university has been granted full freedom of publication of the results from this research programme. Nils Magerøy has been reimbursed by the research programme for attending conferences. Nils Magerøy and Bente E. Moen have been paid by the Royal Norwegian Navy for giving 2–3 lectures annually at courses being arranged by the Royal Norwegian Navy.

## Authors' contributions

All authors together developed the aim and analytical approach for this paper.

NM undertook the statistical analyses, and NM and TM wrote the first draft of the paper.

All authors read and approved the final manuscript.

## Pre-publication history

The pre-publication history for this paper can be accessed here:


